# A ^99m^Tc-Labelled Tetrazine for Bioorthogonal Chemistry. Synthesis and Biodistribution Studies with Small Molecule *trans*-Cyclooctene Derivatives

**DOI:** 10.1371/journal.pone.0167425

**Published:** 2016-12-09

**Authors:** Alyssa Vito, Hussain Alarabi, Shannon Czorny, Omid Beiraghi, Jeff Kent, Nancy Janzen, Afaf R. Genady, Salma A. Alkarmi, Stephanie Rathmann, Zoya Naperstkow, Megan Blacker, Lisset Llano, Paul J. Berti, John F. Valliant

**Affiliations:** 1 Department of Chemistry and Chemical Biology, McMaster University, Hamilton, Ontario, Canada; 2 Department of Chemistry, Faculty of Science, Tanta University, Tanta, Egypt; 3 Centre for Probe Development and Commercialization, McMaster University, Hamilton, Ontario, Canada; 4 Department of Biochemistry & Biomedical Sciences, McMaster University, Hamilton, Ontario, Canada; Purdue University, UNITED STATES

## Abstract

A convenient strategy to radiolabel a hydrazinonicotonic acid (HYNIC)-derived tetrazine with ^99m^Tc was developed, and its utility for creating probes to image bone metabolism and bacterial infection using both active and pretargeting strategies was demonstrated. The ^99m^Tc-labelled HYNIC-tetrazine was synthesized in 75% yield and exhibited high stability *in vitro* and *in vivo*. A *trans*-cyclooctene (TCO)-labelled bisphosphonate (TCO-BP) that binds to regions of active calcium metabolism was used to evaluate the utility of the labelled tetrazine for bioorthogonal chemistry. The pretargeting approach, with ^99m^Tc-HYNIC-tetrazine administered to mice one hour after TCO-BP, showed significant uptake of radioactivity in regions of active bone metabolism (knees and shoulders) at 6 hours post-injection. For comparison, TCO-BP was reacted with ^99m^Tc-HYNIC-tetrazine before injection and this active targeting also showed high specific uptake in the knees and shoulders, whereas control ^99m^Tc-HYNIC-tetrazine alone did not. A TCO-vancomycin derivative was similarly employed for targeting *Staphylococcus aureus* infection *in vitro* and *in vivo*. Pretargeting and active targeting strategies showed 2.5- and 3-fold uptake, respectively, at the sites of a calf-muscle infection in a murine model, compared to the contralateral control muscle. These results demonstrate the utility of the ^99m^Tc-HYNIC-tetrazine for preparing new technetium radiopharmaceuticals, including those based on small molecule targeting constructs containing TCO, using either active or pretargeting strategies.

## Introduction

Recent developments in bioorthogonal coupling chemistry offer a new way to create nuclear molecular imaging and therapy agents by allowing the targeting vector and its radiolabelled coupling partner to be administered separately, in contrast to the traditional method of administering a single molecule containing both the vector and the radiolabel [1]. Pretargeting allows time for the vector to accumulate at the site of interest and clear from non-target organs before injection of a radiolabelled coupling partner that reacts selectively with the targeting vector [2,3]. This approach creates the opportunity to use short half-life isotopes with macromolecular targeting vectors such as antibodies, which typically circulate for long periods of time and clear slowly [4–6]. The approach is also useful for radiometals and small molecule targeting constructs, where direct labelling is often not possible due to a lack of suitable metal-binding functional groups or where the addition of large radiometal-chelate complexes is detrimental to the ability of the conjugate to bind the target of interest.

The challenge of labelling small molecule targeting vectors with radiometals has been a particularly significant issue for targeted technetium radiopharmaceuticals. ^99m^Tc is the most widely used radionuclide in diagnostic medicine; however, the majority of agents used clinically are perfusion type radiopharmaceuticals [7–9]. There are a limited number of examples of targeted Tc agents, largely because of the challenge in mitigating the influence of Tc-chelate complexes on targeting constructs [10]. Pretargeting and bioorthogonal chemistry may offer a means of overcoming these barriers.

One particularly effective pretargeting method involves the inverse electron demand Diels-Alder (IEDDA) reaction between tetrazines and *trans*-cyclooctene (TCO) [11–13]. This system offers a number of advantages including biocompatibility, modest prosthetic group size, and rapid coupling kinetics. Herein we report the use of the IEDDA reaction with a ^99m^Tc-labelled tetrazine and two TCO-derived small molecules; one that targets bone, and a second that is designed to target sites of bacterial infection. These studies demonstrate the utility of the IEDDA reaction for developing new technetium radiopharmaceuticals with small molecule targeting constructs using both active targeting and pretargeting strategies.

## Materials and Methods

### General Procedures

Unless otherwise stated, all chemical reagents were purchased and used as received from Sigma-Aldrich, without further purification. Solvents were purchased from Caledon. ^1^H and ^13^C NMR spectra were recorded on Bruker AV 200 or AV 600 spectrometers. ^1^H NMR chemical shifts are reported in ppm relative to the residual proton signal of the NMR solvents. Coupling constants (*J*) are reported in Hertz (Hz). ^13^C NMR chemical shifts are reported in ppm relative to the carbon-13 signal of the NMR solvents. High resolution mass spectra (HRMS) were obtained on a Waters QToF Ultima Global spectrometer. 99m-Pertechnetate [^99m^TcO_4_]^-^ was obtained in saline solution from a ^99^Mo/^99m^Tc generator supplied by Lantheus Medical Imaging.

Reverse phase analytical HPLC was performed using a Waters 2489 HPLC equipped with a Waters 2489 UV/Vis (λ = 254 nm or 350 nm) and a Bioscan flow-count gamma detector (model 106). Spectra were recorded and processed on Empower 2 software (Waters). Semi-preparative HPLC was performed using a C18, reverse-phase Phenomonex Gemini column (250 × 10 mm, 5 μm) with HPLC grade water (solvent A) and acetonitrile (solvent B) used as eluents. The flow rate was 4 mL/min using a gradient of 95% to 5%; solvent A over 30 min. Analytical HPLC was performed using a C18 reverse-phase Phenomonex column (25 × 4.60 mm, 5 micron) with HPLC grade water containing 0.1% trifluoroacetic acid (TFA) (Solvent A) and acetonitrile containing 0.1% TFA (Solvent B) as eluents. The flow rate was 1 mL/min using a gradient of 95% to 5% solvent A over 30 min. Kinetics studies were conducted on a Cary 50 UV/Vis spectrometer. Radioactivity for biodistribution studies was measured using a Perkin Elmer Wizard 1470 gamma counter.

Animal studies were approved by the Animal Research Ethics Board at McMaster University in accordance with Canadian Council on Animal Care (CCAC) guidelines. Balb/c and CD1 female mice (5 to 6 weeks old) were purchased from Charles River Laboratories and were maintained under Clean/Biohazard Level 2 conditions with 12 h light/dark cycles and given food and water *ad libitum*. Animals were housed for 1 week after arrival to acclimatize prior to initiation of any studies. No animals died during any of the biodistribution studies, prior to sacrifice at the endpoints. Mice were monitored at 4 h and 20 h post injection of bacteria. There was no indication of pain or distress at those times, although a few animals indicated discomfort in the infected leg at the time of sacrifice.

### Synthesis of Boc-protected HYNIC-tetrazine 2

The synthesis of Boc-protected HYNIC-tetrazine was adapted from a procedure reported by Zeglis and coworkers [5]. 3-(4-Benzylamino)-1,2,4,5-tetrazine (13 mg, 0.06 mmol) was dissolved in DMF (2 mL) and DIPEA (21 μL, 0.12 mmol) added. After 15 min of stirring at RT, the pink solution was added to a second premixed solution of 6-Boc-hydrazinopyridine-3-carboxylic acid (**1**, 30 mg, 0.12 mmol) and PyBOP (84 mg, 0.16 mmol) in DMF (2 mL). The combined solutions were stirred overnight under argon. The reaction mixture was concentrated to dryness under reduced pressure and the resulting solid dissolved in a minimum amount of ethyl acetate. The desired product **2** was isolated as a pink solid by silica gel chromatography using an ethyl acetate:hexane mixture (80:20 v/v) as the eluent (16 mg, 65%). ^1^H NMR (600 MHz, Methanol-*d*_4_) δ 10.31 (s, 1H), 8.62 (dd, *J* = 2.3, 0.8 Hz, 1H), 8.59–8.50 (m, 2H), 8.06 (dd, *J* = 8.8, 2.4 Hz, 1H), 7.69–7.54 (m, 2H), 6.73 (dd, *J* = 8.7, 0.9 Hz, 1H), 4.68 (s, 2H), 1.49 (s, 9H) ^13^C NMR (600 MHz, CD_3_OD): 169.3, 168.5, 164.2, 160.1, 159.2, 149.8, 146.7, 139.2, 133.1, 130.2, 123.0, 107.7, 82.73, 45.0, 29.4; HRMS (ESI^+^): for C_20_H_23_N_8_O_3_ calculated: 423.1815 found: 423.1886. HPLC R_t_ = 10 min.

### Reaction kinetics

Kinetic measurements were carried out in triplicate under pseudo-first order conditions using a fixed amount of tetrazine (10^−4^ M) in methanol. The disappearance of the tetrazine absorption at 534 nm was measured at room temperature immediately upon the addition of (*E*)-cyclooct-4-enol (TCO-OH). Studies were repeated using different concentrations of TCO-OH (10 mM, 15 mM and 20 mM) and the second order rate constant determined (S1 File).

### Radiolabelling HYNIC-Tetrazine 3 with ^99m^Tc

Compound **2** (15 mg, 0.036 mmol) was dissolved in 50% v/v TFA:DCM and the mixture stirred over ice for 2 h before being concentrated to dryness. The resulting solid **3** was dissolved in 2:1 v/v DCM:MeOH and transferred to eppendorf tubes prior to evaporation under a stream of nitrogen. A sample of **3** (100 μg, 310 nmol) was subsequently dissolved in water (100 μL) prior to the addition of tricine (500 μL, 100 mg / mL in water), 500 μL ^99m^TcO_4_^-^ (740 MBq) and 10 μL of stannous chloride dihydrate (3 mg / mL in EtOH). The solution was vortexed for 2 min and heated at 60°C for 30 min, at which time the reaction was allowed to cool to RT and the desired product **4** isolated by semi-preparative HPLC. HPLC R_t_ = 13 min; Radiochemical yield = 75%.

### *In vitro* stability

Stability studies were performed following isolation of **4** by semi-preparative HPLC, where the product was first dried using a Biotage V10 evaporation system and the residue reconstituted in 0.9% saline (5.55 MBq / mL saline). Samples were incubated at 37°C and then evaluated by analytical HPLC at various time points over 4 h (S2 File).

### Biodistribution of 4

Biodistribution studies were performed using 4–8 week old female CD1 mice (Charles River Laboratories, Montreal, PQ). Mice were administered approximately 0.54 MBq of **4** (100 μL in 0.9% NaCl) by tail vein injection and sacrificed at t = 0.5, 1, 2 and 6 h post injection (*n* = 3 per time point). Decay correction was used to normalize activity measurements to time of dose preparation for data calculations. Following isolation of tissues and fluids, the mean percent injected dose per gram (%ID/g) and SEM for each sample were calculated.

### Preparation of 5

Following isolation by HPLC and evaporation to dryness using a Biotage evaporator, compound **4** was formulated in 0.9% saline (0.37 MBq / 100 μL) containing TCO-BP (20 mg / kg) and incubated at room temperature for 15 min prior to administration.

### Biodistribution of 4 and TCO-BP

For pretargeting, a 5 mg/mL solution of TCO-BP in saline (100 μL) was administered (20 mg / kg) by tail-vein injection to female Balb/c mice 1 h prior to injection of 0.6 MBq of **4** (100 μL). For studies involving the active targeting complex **5**, a saline solution of the product also administered by tail vein injection. At 6 h post-injection of the labelled compounds, animals were anesthetized with 3% isoflurane and euthanized by cervical dislocation. Fluids, bone (knee, shoulder), and select tissues were collected, weighed, and counted in gamma counter. Decay correction was used to normalize activity measurements to time of dose preparation for data calculations. Data is expressed as %ID/g.

### Synthesis of TCO-vancomycin 6

Preparation of the TCO-vancomycin derivative was performed following the procedure reported by Weissleder and coworkers [14].

### Preparation of active targeting agent 7

Following isolation by HPLC and evaporation to dryness using a Biotage evaporator, compound **4** (0.37 MBq / 100 μL saline) was added to 1 μM **6** in phosphate-buffered saline (PBS) containing 1% fetal bovine serum (FBS). The mixture was left at room temperature for 15 min prior to initiation of biodistribution studies.

### *In vitro* bacteria binding assay

An aliquot of *Staphylococcus aureus* (*S*. *aureus*, ATCC 25923) culture was added to 30 mL of lysogeny broth (LB) in a sterile 250 mL baffled flask. The culture was grown in a shaker incubator at 37°C and 300 rpm for 16 to 24 h until bacteria reached log phase of growth. Bacterial suspensions were aliquoted into 1.5 mL microtubes and centrifuged for 2 min at 11,400 × g. The pellet was washed twice with LB and resuspended in LB to an OD_600_ of 2.0. Each 1.5 mL sample was transferred to new 1.5 mL microtubes and centrifuged for 2 min at 11,400 × g. The supernatant was discarded and pellets resuspended in 1.5 mL LB containing either 20 μM **6** or 20 μM **6** + 200 μM vancomycin (each done in triplicate). Each sample was transferred into a separate 5 mL round-bottom tube and placed in a shaking incubator for 30 min, at 37°C. Following pre-incubation with vancomycin-TCO, samples were washed twice with LB and resuspended in 1.5 mL of LB containing either **4** (7.4 Bq / μL) alone or the same amount of **4** in the presence of 200 μM vancomycin. Each sample was transferred to a new 5 mL round-bottom incubation tube and cultures were placed in a shaker incubator at 37°C and 300 rpm. At 1 and 6 h following addition of **4**, a 500 μL aliquot from each sample was taken and cells were pelleted by centrifuging for 2 min at 11,400 × g. A 480 μL aliquot of each supernatant was transferred to a separate plastic RIA test tube (Perkin Elmer, 1270–401). Pellets were washed once with PBS (500 μL each), and centrifuged as described above. Supernatant (500 μL) was collected and combined with supernatant previously collected for that pellet. Each pellet was resuspended in 400 μL of 5% SDS in water and incubated for 30 to 45 min at RT. A 300 μL aliquot of each pellet lysate was collected in a separate RIA test tube. Pellets and supernatants were then counted using a gamma counter. Data were analyzed and expressed as percent of **4** bound to *S*. *aureus* compared to total activity added.

### Preparation of *Staphylococcus aureus* inoculum

Several 3 mL overnight cultures of *S*. *aureus* (ATCC 25923) were grown in LB at 37°C and shaking at 300 rpm. Bacterial cultures were pooled and cells were pelleted at 11,400 × g for 2 min. Cells were washed with sterile PBS (Invitrogen, Mississauga ON) and centrifuged again at 11,400 × g for 2 min. The pellet was resuspended and further diluted with sterile PBS to an appropriate concentration prior to inoculation (20 ×10^8^ CFU/mL).

### Biodistribution Studies in mice with *S*. *aureus* infection

Female Balb/c mice (7 to 8 weeks old) were administered *S*. *aureus* inoculum into the right calf muscle (50 μL; 1×10^8^ CFU/injection), 24 h before injection of the labelled compound. For the active targeting study, mice were administered ~0.30 MBq of **7** (200 μL in PBS, 1% FBS) via tail vein injection. For the pretargeting study, 1.2 mg/kg of **6** was administered 1 h prior to **4**. For all studies, mice were anesthetized with 3% isoflurane, euthanized by cervical dislocation, and tissue and fluid samples were collected at 1 h and 6 h post injection of the labeled compound (n = 3 per time point). The left (control) and right (infected) calf muscles and all other tissues and fluids were collected, weighed and counted using a gamma counter. Decay correction was used to normalize activity measurements to time of dose preparation and data reported as %ID/g.

## Results and Discussion

### Synthesis of the tetrazine ligand 2

**Fig 1:**
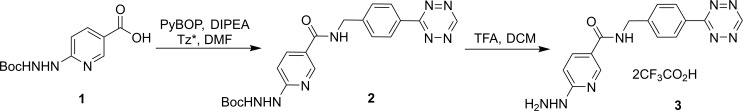
Synthesis scheme for the preparation of 3. A protected form of HYNIC (**1**) was coupled to a commercially available tetrazine to form **2**. The Boc group was removed prior to labelling by treatment with TFA in DCM to produce **3**. Tz* = (4-(1,2,4,5-tetrazin-3-yl)phenyl) methanamine.

There is only one report of a ^99m^Tc labelled tetrazine that was prepared using a hydrazinonicotinic acid (HYNIC) ligand [15]. We similarly employed a HYNIC ligand, but in place of a methyl substituted tetrazine we opted to use the more reactive and commercially available reagent (4-(1,2,4,5-tetrazin-3-yl)phenyl)methanamine (Fig 1). This particular tetrazine was selected because of its high reactivity towards TCO and proven effectiveness for *in vivo* IEDDA reactions [16]. The target ligand was prepared by coupling the commercial tetrazine with the Boc-protected HYNIC precursor **1** [17] in the presence of pyBOP in DMF. Isolation of the pink product **2** was achieved by silica gel chromatography in 65% yield.

### Kinetic studies

The kinetics of the reaction between **2** and (*E*)-cyclooct-4-enol (TCO-OH) were followed spectrophotometrically by monitoring the decrease in absorbance at 534 nm as a function of time. The reaction was complete in seconds (S1 File), with a second order rate constant of 92 ± 6 M^-1^s^-1^ in methanol. This was similar to that of the parent tetrazine (82 ± 2 M^-1^s^-1^ in methanol) measured in head-to-head studies.

### Radiolabelling with ^99m^Tc

**Fig 2:**
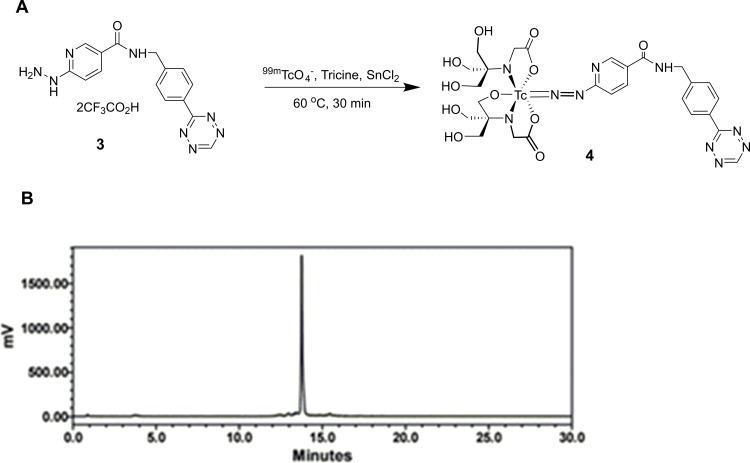
Radiolabelling scheme for the HYNIC-tetrazine ligand 3 and HPLC chromatogram of the isolated product 4. ^99m^Tc labelling of the HYNIC-tetrazine **3** (A). A γ-HPLC chromatogram of the purified final product **4** (B).

Prior to labelling, the Boc groups were removed from **2** by treatment with TFA in DCM (Fig 1). Labelling of **3** with ^99m^Tc involved treatment with ^99m^TcO_4_^-^ and SnCl_2_ in the presence of a co-ligand (Fig 2) [18]. The co-ligand serves to occupy vacant coordination sites and can be easily varied, providing a convenient means of tuning the pharmacokinetics of the metal complex [19]. Tricine was selected here as the coligand as it has been shown to be highly effective at stabilizing Tc-HYNIC complexes and minimizing non-specific binding *in vivo* [20]. Optimizing the labelling conditions involved varying the reaction temperature, time, pH and the amount of **3**. The reaction yield was particular sensitive to the amount of tetrazine ligand used where 100 μg was optimal. Ultimately, the desired product **4** was obtained in 75% radiochemical yield and 98% radiochemical purity (Fig 2) when the reaction was run at 60°C for 30 min. Because the synthesis was performed directly from ^99m^TcO_4_^-^, which is the chemical form of technetium that is eluted from ^99^Mo/^99m^Tc generators, the product can be produced easily, making it readily accessible and translatable for use in clinical studies.

### *In vitro* stability and TCO reactivity

The stability of **4** in 0.9% saline was assessed over 4 h by HPLC and showed minimal decomposition (S2 File). The reactivity of the labeled tetrazine with TCO-OH was tested subsequently. γ-HPLC analysis showed rapid disappearance of the peak associated with **4** and appearance of multiple peaks (S3 File) associated with the formation of the tautomeric and diastereomeric isomers of dihydropyridazine and pyridazine [21].

### Biodistribution of 4

**Fig 3:**
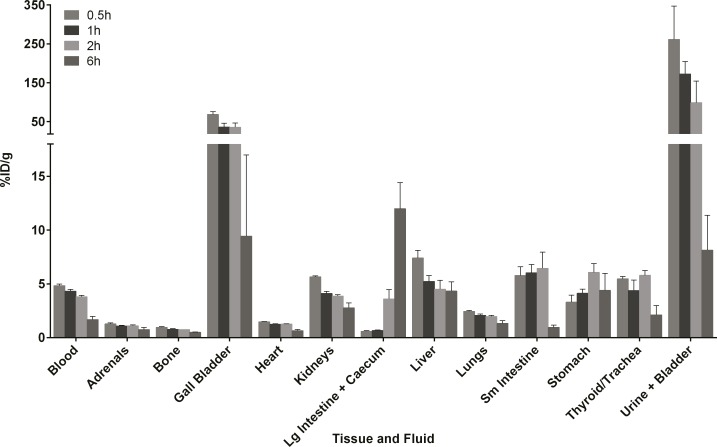
Biodistribution data for 4. Data are presented as the mean (± SEM) percent injected dose per gram (%ID/g) for selected tissues and fluids from CD1 mice at 0.5, 1, 2 and 6 h post injection (n = 3 per time point). Approximately 0.88 MBq were administered per mouse. Full biodistribution data can be found in the supporting information (S4 File).

The biodistribution of the ^99m^Tc-HYNIC-tetrazine **4** alone was evaluated in healthy CD1 mice at multiple time points. Compound **4** showed modest levels in the blood at 0.5 h (4.8 ± 0.1% injected dose (ID)/g) which ultimately decreased to 1.7 ± 0.3%ID/g at 6 h (Fig 3). Activity was observed in the stomach and thyroid, indicating the presence of pertechnetate. It is worth noting that the levels in these organs did not increase over 6 h, suggesting that the compound was not continually degraded during the timeframe of the study. The product showed significant uptake in the gall bladder that decreased significantly by 6 h post-injection. In addition, **4** was detected in multiple organs and tissues, which suggests that the labelled tetrazine should be available to react with TCO-conjugates *in vivo*. These results are consistent with the biodistribution of other HYNIC-tricine derivatives [22–25] and follows a similar trend to that recently reported for the analogous complex derived from a methylated tetrazine derivative [15,26].

### Targeting bone using a TCO-bisphosphonate (TCO-BP) and 4

**Fig 4:**
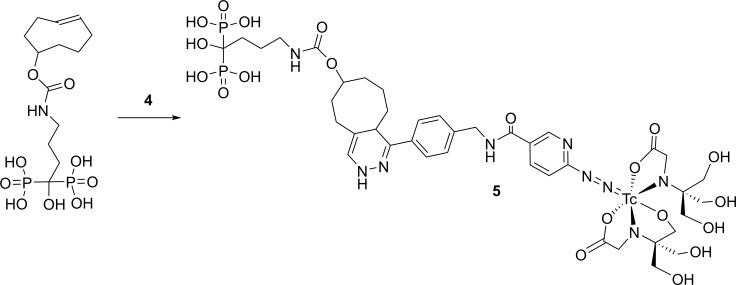
Synthesis scheme for the actively targeted derivative, ^99m^Tc-HYNIC-tetrazine-TCO-BP (5). The TCO derivative of the bisphosphonate, alendronate (TCO-BP) was mixed with 4 prior to administration to mice.

The next step was to test the ability of **4** to react with TCO derivatives *in vivo*. To this end a TCO-bisphosphonate derivative (TCO-BP) was employed. Bisphosphonates bind to regions of calcium accretion in bone and have been used as radiometal binding ligands and in routine clinical imaging of skeletal injuries, when labelled with ^99m^Tc [27–29]. Since it can target turnover in normal bone in mice, which is typically found in shoulder and knee joints, and is easily prepared in a single step, TCO-BP is a convenient reagent to evaluate new radiolabelled tetrazines *in vivo*. To this end, a solution of TCO-BP in saline was administered i.v. to Balb/c mice (20 mg/kg), 1 h prior to injection of **4**. For comparison, an actively targeted construct was prepared by combining **4** with TCO-BP to produce the ^99m^Tc-HYNIC-tetrazine-TCO-BP complex **5**, prior to administration (Fig 4). For both biodistribution studies, mice were euthanized and tissues collected 6 h post-injection. The radioactivity in different tissues and fluids was determined and % ID/g calculated.

**Fig 5:**
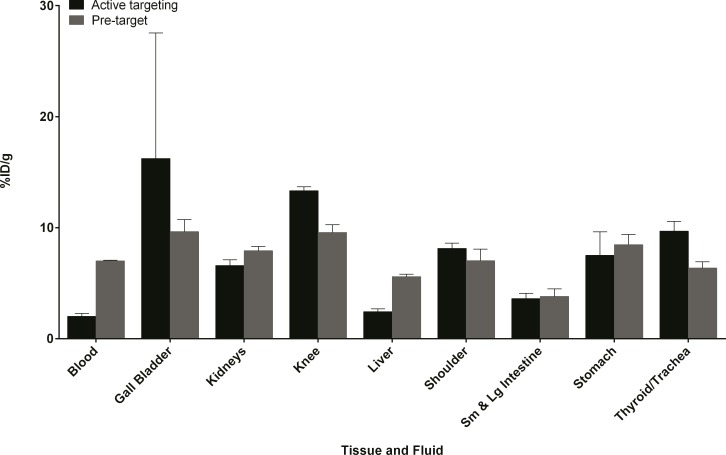
Biodistribution data comparing active targeting to pretargeting. Active targeting with ^99m^Tc-HYNIC-tetrazine-TCO-BP (**5**) (black bars) is compared to pretargeting of TCO-BP administered 1 h prior to **4** (gray bars). Data are expressed as the mean (± SEM) %ID/g for selected tissues and fluids from Balb/c mice (n = 3 per time point). Tabulated biodistribution data can be found in the supporting information (S4 File).

For pretargeting (Fig 5), there was significant uptake in regions of active bone metabolism, mainly the knee (9.6 ± 0.7%ID/g) and shoulder (7.0 ± 1%ID/g). This was significantly higher than that for the control in which **4** was given alone (S4 File), where uptake was < 1%ID/g in bone at all time points. The actively targeted construct **5** also showed significant uptake in the knee (13.3 ± 0.4%ID/g) and shoulder (8.1 ± 0.5%ID/g). Compound **5** exhibited lower radioactivity concentrations in the blood and liver, which is likely due to the increased polarity of the bisphosphonate conjugate compared to the more hydrophobic parent tetrazine. The data clearly demonstrates that compound **4** can react with TCO derivatives *in vivo*, and that the IEDDA reaction is a convenient way to prepare actively targeted constructs.

### Targeting infection using TCO-vancomycin

**Fig 6:**
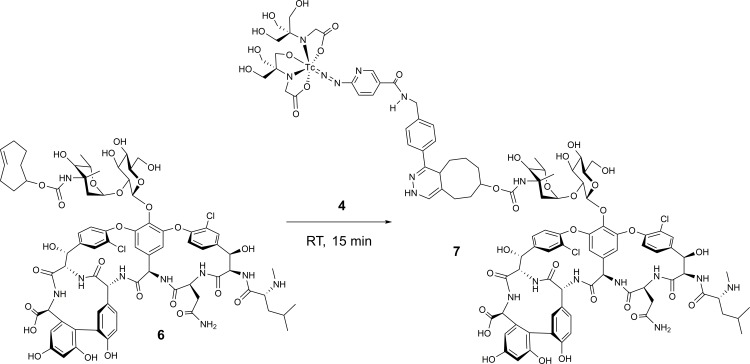
Synthesis scheme for ^99m^Tc-HYNIC-tetrazine-TCO-vancomycin (7) from TCO-vancomycin (6) and 4.

Having demonstrated the ability to localize **4** to bone using pretargeting and active targeting with TCO-BP, we next tested a TCO-vancomycin derivative as means of localizing **4** to sites of infection. There has been a longstanding search for Tc-radiopharmaceuticals for detecting bacterial infection [30,31]. One approach has been to radiolabel antibiotics directly with ^99m^Tc [30]. This met with limited success largely due to lack of stability of the metal complex *in vivo* and the detrimental influence coordination of the antibiotic to ^99m^Tc had on delivery and binding to the infection. One approach to overcoming these issues is to use TCO-derivatized antibiotics, pretargeting and a ^99m^Tc-labelled tetrazine. As was mentioned, the same chemistry can be used to generate actively targeted radiopharmaceuticals. To these ends, TCO-vancomycin (**6**), which has been used to image bacteria *in vitro* [14], was employed as a model targeting molecule along with **4** to create new radiopharmaceuticals for imaging infection using both active and pretargeting strategies (Fig 6).

**Fig 7:**
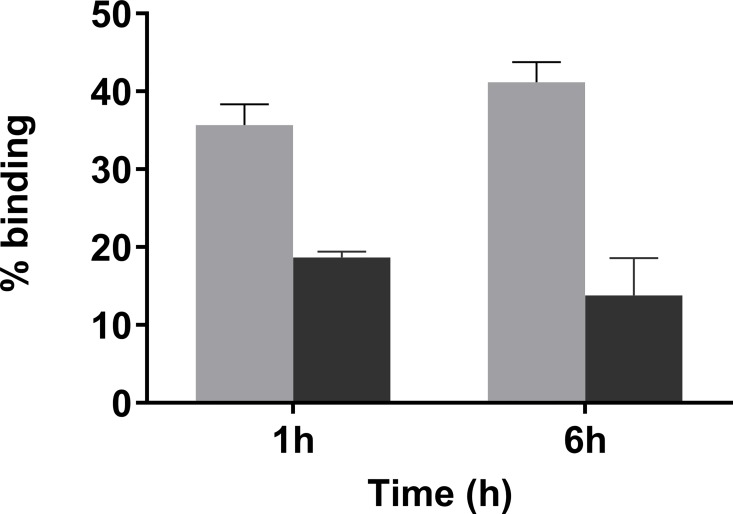
Binding of 4 to *S*. *aureus in vitro* using TCO-vancomycin 6. *S*. *aureus* were pretreated with **6** in the absence (gray) or presence (black) of a 10-fold excess of vancomycin. The mean percentages (± SEM) of total radioactivity bound after 1 and 6 h incubation times with **4** are shown.

Initial *in vitro* studies were performed with *S*. *aureus* that was treated with **6** for 30 min in the presence or absence of vancomycin as a blocking agent. The amount of **6** used and incubation time were below that which would is known to have a bactericidal effect [14]. Following a washing step to remove unbound **6**, compound **4** was added and the samples incubated at 37°C for 1 and 6 h prior to washing. At both time points there was significantly more activity in the samples containing **6** alone compared to those containing **6** and excess vancomycin. At 6 h, the amount of **4** bound to *S*. *aureus* was 2.5-fold higher than the control containing the blocking agent (Fig 7). These results are consistent with prior reports, which showed that **6** binds specifically to vancomycin binding sites on bacteria, and that it maintains reactivity towards tetrazines when bound to bacteria [14].

### *In vivo* testing of 4 to target sites of bacterial infection.

**Fig 8:**
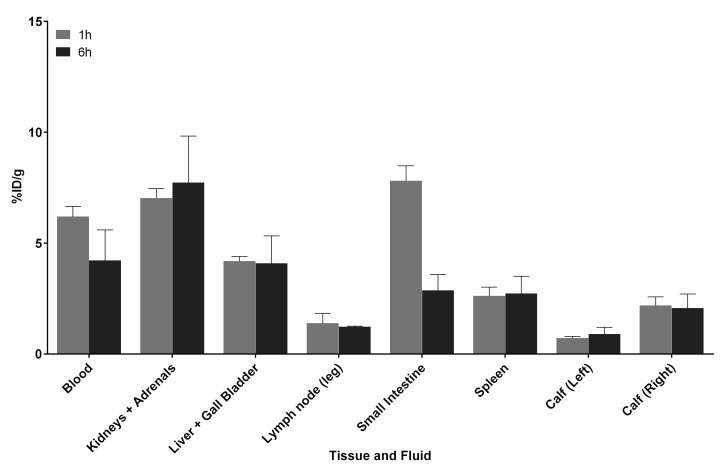
Biodistribution data for active targeting of *S*. *aureus* infection using ^99m^Tc-HYNIC-tetrazine-TCO-vancomycin (7). Compounds **4** and **6** were combined prior to i.v. injection of Balb/c mice (n = 3 per time point). Select fluids and tissues were collected at 1 (gray bars) and 6 h (black bars) post injection, including the infected calf muscle (right), and the non-infected calf muscle (left). Data are expressed as the mean percent injected dose per gram (%ID/g) ± SEM. Tabulated biodistribution data can be found in the supporting information (S4 File).

Compounds **4** and **6** were evaluated for their ability to target sites of infection *in vivo* using active and pretargeting approaches. Infections were created by administering *S*. *aureus* to the right calf muscle in Balb/c mice, with the non-injected left calf muscle as the contralateral control. Similar infection models has been used extensively to evaluate bacterial infection imaging agents [32–37]. For the active targeting study, **4** was combined with **6** (Fig 7) and injected i.v. (tail vein), within 15 min. Tissues and fluids were collected at 1 and 6 h post-injection to determine the %ID/g (Fig 8). The infected calf muscle showed a 3-fold increase in radioactivity at 1 h compared to the control muscle. The radioactivity in the infected calf remained constant over 6 h post-injection (2.2 ± 0.4%ID/g at 1 h and 2.1 ± 0.6%ID/g at 6 h). The spleen also showed some accumulation of radioactivity that remained over the duration of the study (2.6 ± 0.4%ID/g at 1 h and 2.7 ± 0.8%ID/g at 6 h).

**Fig 9:**
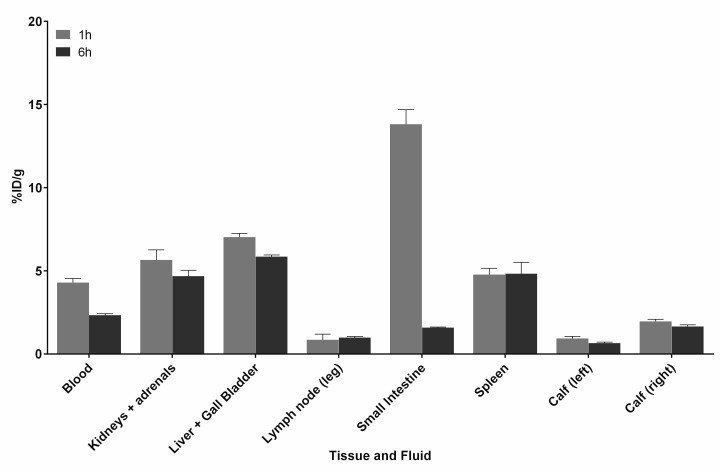
Biodistribution data for pretargeting using TCO-vancomycin (7) and 4. Balb/c mice (n = 3 per time point) were given *S*. *aureus* infections in the right calf muscle, and 24 h later were treated with **7** administered 1 h prior to **4**. Radioactivity was measured 1 and 6 h later in selected tissues and fluids. Data are expressed as the mean percent injected dose per gram (%ID/g) ± SEM. Tabulated biodistribution values can be found in the Supporting Information.

Results from pretargeting studies (Fig 9) showed a 2-fold higher radioactivity concentration localized to infected versus non-infected control muscle at 1 h (2.0 ± 0.1%ID/g versus 0.9 ± 0.1%ID/g, respectively), increasing to 2.5-fold higher localization at 6 h post injection (1.7 ± 0.1%ID/g and 0.7 ± 0.1%ID/g, respectively). As with the active targeting study, accumulation within the spleen occurred and remained consistent over the duration of the study (4.8 ± 0.4%ID/g at 1 h and 4.8 ± 0.7%ID/g at 6 h). The pretargeting studies show that the ^99m^Tc-labelled HYNIC derivative can localize preferentially to sites of infection.

## Conclusions

A new Tc-labelled tetrazine was prepared and shown to react *in vivo* via the IEDDA reaction with two TCO-derived small molecules; one targeting regions of bone metabolism, and the second *S*. *aureus* infections. While pretargeting has the disadvantage of requiring administration of two compounds, it does offer a new approach for labelling small molecules with the most widely used radionuclide in diagnostic medicine. Furthermore, with access to **4**, the high yielding and selective IEDDA reaction can also be used to prepare actively targeted Tc radiopharmaceuticals from small molecules that do not have the appropriate donors or arrangement of heteroatoms to bind the radiometal directly.

## Supporting Information

S1 File Reaction kinetics and rate constant calculation for HYNIC-tetrazine (2) with TCO-OH. Fig A: Absorbance at 534 nm (A_534_) versus time following the addition of TCO-OH (10 mM) to **2** (1.0 mM) in MeOH. Fig B: Plots of *k*_obs_ versus TCO concentration for the reaction of **2** with TCO-OH (A), and reaction of **1** with TCO-OH (B) in MeOH
(PDF)

S2 File Stability study of ^99m^Tc-HYNIC-tetrazine (4) in saline. Fig C: γ-HPLC chromatograms of **4** following incubation in saline at 0.5 h (top) and 4 h (bottom).
(PDF)

S3 File Reaction products of ^99m^Tc-HYNIC-tetrazine (4) with TCO-OH. Fig D: γ-HPLC chromatograms of **4** alone (B) or 0.5 h after reaction of **4** with TCO-OH (A).
(PDF)

S4 File Biodistribution data. Table A: Biodistribution data for **4**. Table B: Biodistribution data for active targeting with **5**. Table C: Biodistribution data for pretargeting with TCO-BP and **4**. Table D: Biodistribution data for active targeting with ^99m^Tc-HYNIC-tetrazine-TCO-vancomycin.
(PDF)
