# Correction: A ^99m^Tc-Labelled Tetrazine for Bioorthogonal Chemistry. Synthesis and Biodistribution Studies with Small Molecule *trans*-Cyclooctene Derivatives

**DOI:** 10.1371/journal.pone.0171909

**Published:** 2017-02-06

**Authors:** Alyssa Vito, Hussain Alarabi, Shannon Czorny, Omid Beiraghi, Jeff Kent, Nancy Janzen, Afaf R. Genady, Salma A. Al-Karmi, Stephanie Rathmann, Zoya Naperstkow, Megan Blacker, Lisset Llano, Paul J. Berti, John F. Valliant

The eighth author’s name is spelled incorrectly. The correct name is: Salma A. Al-Karmi.
