# Supplementary material for: A 99mTc-Labelled Tetrazine for Bioorthogonal Chemistry. Synthesis and Biodistribution Studies with Small Molecule trans-Cyclooctene Derivatives
Source: PLoS One. 2016 Dec 9;11(12):e0167425. doi: 10.1371/journal.pone.0167425 (PMC5147877; doi:10.1371/journal.pone.0167425)
Supplement: S1 File — Fig A: Absorbance at 534 nm (A534) versus time following the addition of TCO-OH (10 mM) to 2 (1.0 mM) in MeOH. Fig B: Plots of kobs versus TCO concentration for the reaction of 2 with TCO-OH (A), and reaction of 1 with TCO-OH (B) in MeOH (PDF) [file pone.0167425.s001.pdf]

Reaction kinetics and rate constant calculation for HYNIC-tetrazine (2) with TCO-OH.

Fig A:

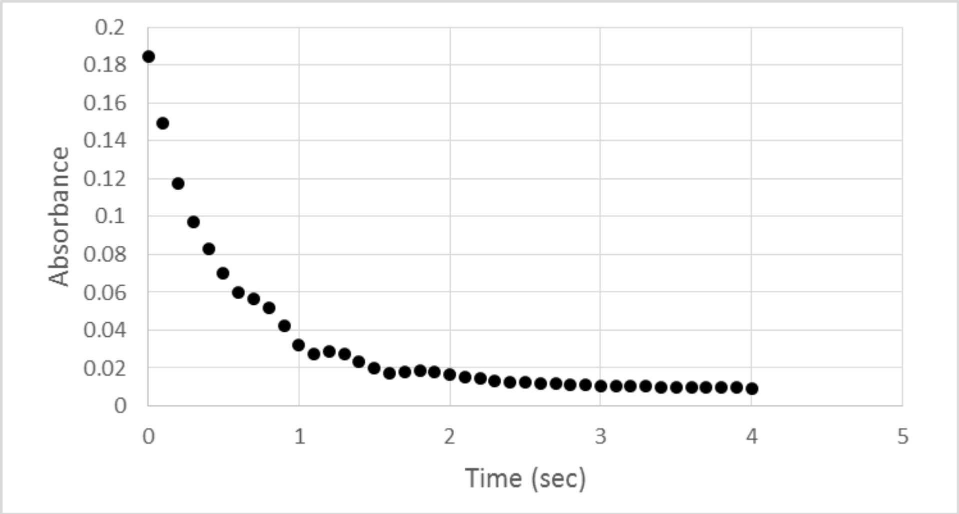

Absorbance at 534 nm (A_{534}) versus time following the addition of TCO-OH (10 mM) to **2** (1.0 mM) in MeOH.

Fig B:

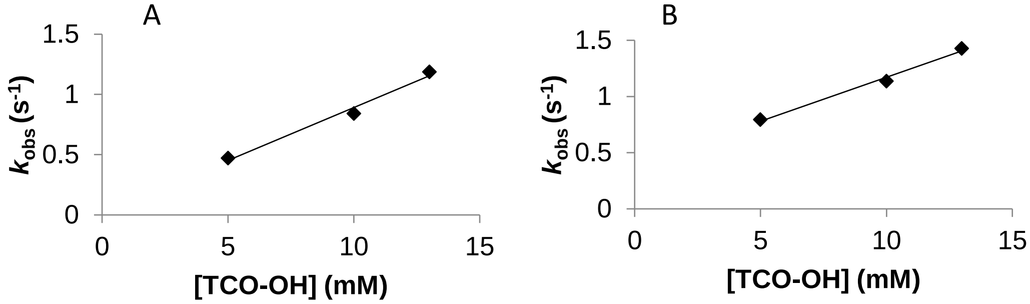

Plots of k_{obs} versus TCO concentration for the reaction of **2** with TCO-OH (A), and reaction of **1** with TCO-OH (B) in MeOH
