# Supplementary material for: A 99mTc-Labelled Tetrazine for Bioorthogonal Chemistry. Synthesis and Biodistribution Studies with Small Molecule trans-Cyclooctene Derivatives
Source: PLoS One. 2016 Dec 9;11(12):e0167425. doi: 10.1371/journal.pone.0167425 (PMC5147877; doi:10.1371/journal.pone.0167425)
Supplement: S2 File — Fig C: γ-HPLC chromatograms of 4 following incubation in saline at 0.5 h (top) and 4 h (bottom). (PDF) [file pone.0167425.s002.pdf]

Stability study of ^{99m}Tc-HYNIC-tetrazine (4) in saline

Fig C:

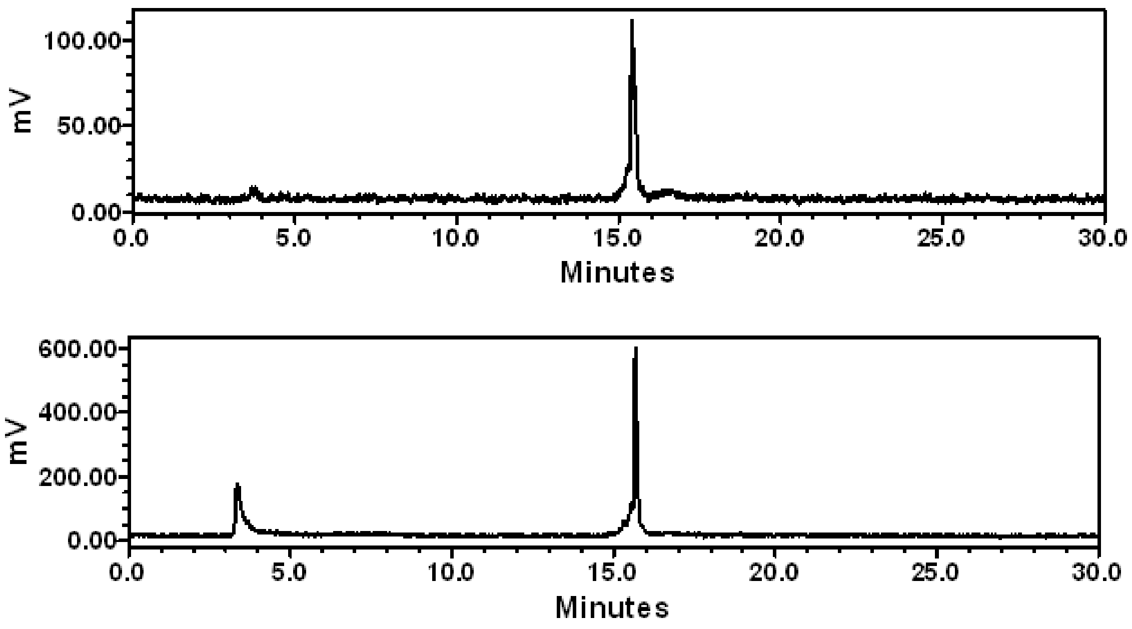

γ-HPLC chromatograms of **4** following incubation in saline at 0.5 h (top) and 4 h (bottom).
