# Supplementary material for: A 99mTc-Labelled Tetrazine for Bioorthogonal Chemistry. Synthesis and Biodistribution Studies with Small Molecule trans-Cyclooctene Derivatives
Source: PLoS One. 2016 Dec 9;11(12):e0167425. doi: 10.1371/journal.pone.0167425 (PMC5147877; doi:10.1371/journal.pone.0167425)
Supplement: S3 File — Fig D: γ-HPLC chromatograms of 4 alone (B) or 0.5 h after reaction of 4 with TCO-OH (A). (PDF) [file pone.0167425.s003.pdf]

Reaction products of ^{99m}Tc-HYNIC-tetrazine (4) with TCO-OH

Fig D:

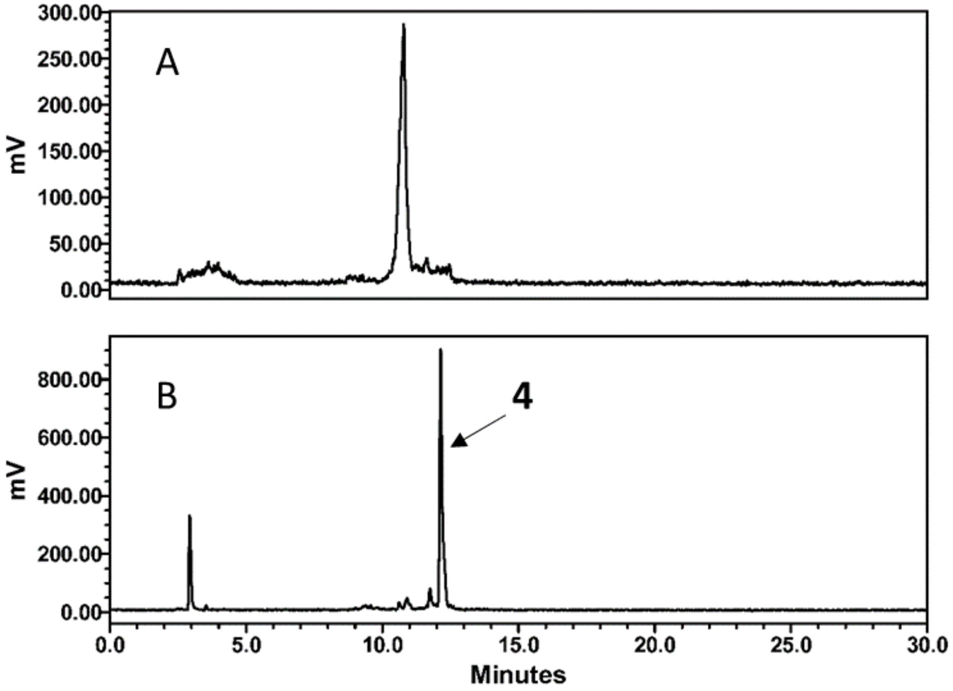

γ-HPLC chromatograms of **4** alone (B) or 0.5 h after reaction of **4** with TCO-OH (A).
