# Supplementary material for: A 99mTc-Labelled Tetrazine for Bioorthogonal Chemistry. Synthesis and Biodistribution Studies with Small Molecule trans-Cyclooctene Derivatives
Source: PLoS One. 2016 Dec 9;11(12):e0167425. doi: 10.1371/journal.pone.0167425 (PMC5147877; doi:10.1371/journal.pone.0167425)
Supplement: S4 File — Table A: Biodistribution data for 4. Table B: Biodistribution data for active targeting with 5. Table C: Biodistribution data for pretargeting with TCO-BP and 4. Table D: Biodistribution data for active targeting with 99mTc-HYNIC-tetrazine-TCO-vancomycin. (PDF) [file pone.0167425.s004.pdf]

Biodistribution data.

Table A: Biodistribution data for **4**.

	Timepoint (h)			
**Organs**	**0.5**	**1**	**2**	**6**
**Blood**	4.85 ± 0.14	4.33 ± 0.17	3.82 ± 0.12	1.70 ± 0.28
**Adipose**	0.35 ± 0.08	0.18 ± 0.02	0.31 ± 0.02	0.17 ± 0.03
**Adrenals**	1.29 ± 0.09	1.09 ± 0.07	1.12 ± 0.08	0.77 ± 0.17
**Bone**	0.97 ± 0.06	0.78 ± 0.07	0.77 ± 0.00	0.51 ± 0.02
**Brain**	0.10 ± 0.01	0.07 ± 0.00	0.07 ± 0.00	0.04 ± 0.01
**Gall Bladder**	68.56 ± 6.86	36.03 ± 9.68	35.90 ± 10.90	9.45 ± 7.53
**Heart**	1.49 ± 0.01	1.26 ± 0.04	1.27 ± 0.03	0.66 ± 0.11
**Kidneys**	5.67 ± 0.08	4.12 ± 0.18	3.88 ± 0.12	2.79 ± 0.44
**Lg Intestine + Caecum**	0.60 ± 0.06	0.69 ± 0.04	3.61 ± 0.87	11.99 ± 2.43
**Liver**	7.42 ± 0.70	5.23 ± 0.54	4.52 ± 0.83	4.35 ± 0.85
**Lungs**	2.46 ± 0.07	2.09 ± 0.12	1.98 ± 0.11	1.34 ± 0.24
**Pancreas**	0.77 ± 0.01	0.61 ± 0.05	0.58 ± 0.04	0.34 ± 0.05
**Skeletal Muscle**	0.53 ± 0.04	0.40 ± 0.01	0.39 ± 0.01	0.33 ± 0.06
**Sm Intestine**	5.79 ± 0.80	6.04 ± 0.76	6.46 ± 1.49	0.96 ± 0.21
**Spleen**	0.92 ± 0.04	0.75 ± 0.05	0.74 ± 0.06	0.54 ± 0.07
**Stomach**	3.31 ± 0.64	4.14 ± 0.38	6.08 ± 0.80	4.41 ± 1.57
**Thyroid/Trachea**	5.48 ± 0.21	4.40 ± 0.97	5.81 ± 0.44	2.13 ± 0.85
**Urine + Bladder**	261.83 ± 85.39	172.71 ± 31.98	98.92 ± 55.68	8.15 ± 3.24

Note: Data are %ID/g, expressed as mean ± SEM.

Table B: Biodistribution data for active targeting with **5**.

Organs	Timepoint (h) 6h		
**Blood**	2.01	±	0.27
**Gall Bladder**	16.24	±	11.32
**Kidneys**	6.59	±	0.52
**Knee**	13.33	±	0.37
**Liver**	2.42	±	0.27
**Shoulder**	8.13	±	0.49
**Sm & Lg Intestine**	3.62	±	0.47
**Stomach**	7.50	±	2.13
**Thyroid/Trachea**	9.68	±	0.90

Note: Data are %ID/g, expressed as mean ± SEM.

Table C: Biodistribution data for pretargeting with TCO-BP and **4**.

Organs	Timepoint (h) 6h		
**Blood**	7.00	±	0.08
**Gall Bladder**	9.65	±	1.08
**Kidneys**	7.92	±	0.39
**Knee**	9.57	±	0.71
**Liver**	5.60	±	0.22
**Shoulder**	7.03	±	1.04
**Sm & Lg Intestine**	3.81	±	0.67
**Stomach**	8.48	±	0.91
**Thyroid/Trachea**	6.37	±	0.55

Note: Data are %ID/g, expressed as mean ± SEM.

Table D: Biodistribution data for active targeting with ^{99m}Tc-HYNIC-tetrazine-TCO-vancomycin.

	Timepoint (h)	
**Organs**	**1**	**6**
**Blood**	6.20 ± 0.46	4.22 ± 1.38
**Kidneys + Adrenals**	7.03 ± 0.43	7.73 ± 2.10
**Liver + Gall Bladder**	4.19 ± 0.21	4.09 ± 1.24
**Lymph node (leg)**	1.39 ± 0.45	1.23 ± 0.03
**Small Intestine**	7.81 ± 0.68	2.87 ± 0.72
**Spleen**	2.62 ± 0.40	2.73 ± 0.78
**Calf (Left)**	0.72 ± 0.08	0.90 ± 0.31
**Calf (Right)**	2.19 ± 0.39	2.07 ± 0.64

Note: Data are %ID/g, expressed as mean ± SEM.

Table E: Biodistribution data for pretargeting with TCO-vancomycin (**7**) with ^{99m}Tc-HYNIC-tetrazine (**4**).

	Timepoint (h)	
**Organs**	**1**	**6**
**Blood**	4.29 ± 0.25	2.33 ± 0.09
**Kidneys + Adrenals**	5.65 ± 0.61	4.68 ± 0.36
**Liver + Gall Bladder**	7.03 ± 0.22	5.86 ± 0.09
**Lymph node (leg)**	0.85 ± 0.34	0.99 ± 0.07
**Small Intestine**	13.82 ± 0.87	1.59 ± 0.03
**Spleen**	4.78 ± 0.38	4.83 ± 0.69
**Calf (Left)**	0.93 ± 0.13	0.65 ± 0.07
**Calf (Right)**	1.96 ± 0.13	1.65 ± 0.10

Note: Data are %ID/g, expressed as mean ± SEM.
